# Multifunctional acyltransferases involved in the synthesis of triacylglycerol, fatty acid phytyl esters and plastoquinol esters in cyanobacteria

**DOI:** 10.1007/s00425-025-04700-6

**Published:** 2025-05-02

**Authors:** Amita Shajil Das, Arpita Shajil Das, Zishuo Chen, Helga Peisker, Katharina Gutbrod, Georg Hölzl, Peter Dörmann

**Affiliations:** Institute of Molecular Physiology and Biotechnology of Plants (IMBIO), University of Bonn, Karlrobert-Kreiten-Str. 13, 53115 Bonn, Germany

**Keywords:** Ester, Lipid, Non-polar, *Gloeobacter*, *Synechococcus*, *Synechocystis*

## Abstract

**Main conclusion:**

The multifunctional acyltransferases (MFAT) from *Synechocystis sp.* PCC 6803 and *Synechococcus sp.* PCC 7002 synthesize triacylglycerol, fatty acid phytyl esters, acylated plastoquinol-9 and acylated plastoquinone C, while *Gloeobacter violaceus* PCC 7421 synthesizes acylated plastoquinol-9 in an MFAT-independent pathway.

**Abstract:**

Cyanobacteria contain large amounts of polar lipids in their thylakoid membranes, but the contents of nonpolar lipids are low. We previously identified triacylglycerol (TAG) and fatty acid phytyl esters (FAPE) in *Synechocystis sp.* PCC 6803, and described a gene (*slr2103*) involved in TAG and FAPE synthesis. Other studies showed that *Synechocystis sp.* PCC 6803 and *Synechococcus sp.* PCC 7002 synthesizes acylated forms of plastoquinol-9 (acyl-PQH) and of plastoquinone C (acyl-PQC), which carries the fatty acid on a hydroxyl group on the isoprenoid chain, but TAG and FAPE were not detected. We confirm here that *Synechocystis sp.* PCC 6803 and *Synechococcus sp.* PCC 7002 contain TAG, FAPE, acyl-PQH and acyl-PQC. Expression of *slr2103* and the related gene *A0918* from *Synechococcus sp.* PCC 7002 in *Escherichia coli,* and analysis of the respective cyanobacterial mutants revealed that the two proteins acylate diacylglycerol, phytol, and the plastoquinol-9 analog decylplastoquinol. Therefore, *slr2103* and *A0918* encode multifunctional acyltransferases (MFAT) with broad substrate specificities. *Gloeobacter violaceus* PCC 7421, a primitive cyanobacterium that lacks an MFAT-like gene, accumulates acyl-PQH, indicating that this strain harbors an MFAT-independent acyltransferase capable of acylating plastoquinol-9. These results demonstrate that cyanobacteria synthesize different nonpolar lipids including TAG, FAPE and acylated forms of plastoquinol, employing MFAT-dependent and MFAT-independent pathways.

**Supplementary Information:**

The online version contains supplementary material available at 10.1007/s00425-025-04700-6.

## Introduction

Cyanobacteria are single-cell prokaryotic organisms that execute oxygenic photosynthesis as found in plants. The photosynthetic complexes are embedded into the thylakoid membranes, which consist of two galactolipids (monogalactosyldiacylglycerol, MGDG; digalactosyldiacylglycerol, DGDG), phosphatidylglycerol and a sulfolipid (sulfoquinovosyldiacylglycerol, SQDG). While the polar lipids are highly abundant in cyanobacteria, the amounts of nonpolar lipids including triacylglycerol (TAG) are extremely low. Under adverse environmental conditions like nitrogen deprivation, carbon in cyanobacteria is mostly stored in the form of polyhydroxybutyrate and glycogen, rather than TAG (Řezanka et al. 2012; Tanaka et al. 2020). We showed previously that *Synechocystis sp.* PCC 6803 (Syn6803) accumulates nonpolar lipids, including TAG and fatty acid phytyl esters (FAPE) (Aizouq et al. 2020). FAPEs are esters of fatty acids with phytol, which is derived from chlorophyll degradation. In plants like *Arabidopsis thaliana*, FAPEs are synthesized by two enzymes, phytyl ester synthases 1 and 2 (PES1, PES2) which belong to the family of esterase/lipase/thioesterases (ELT). Syn6803 contains a gene, *slr2103,* which shows sequence similarity with the acyltransferase domains of PES1 and PES2 (Lippold et al. 2012; Aizouq et al. 2020). Expression of *slr2103* in *Escherichia coli* resulted in the synthesis of TAG and FAPE, while these two lipids were absent from the *Δslr2103* mutant of Syn6803, indicating that slr2103 is involved in TAG and FAPE synthesis (Aizouq et al. 2020).

Additional nonpolar lipids were identified in Syn6803 and other cyanobacteria, including acylated forms of plastoquinol-9 (acyl-PQH), and of a hydroxy-plastoquinone-9 carrying an acyl moiety on a hydroxyl group of the isoprenoid chain (acyl-PQC; PQB) (Ishikawa et al. 2023; Kondo et al. 2023a, b; Mori-Moriyama et al. 2023). Acyl-PQH was also detected in *Nostoc punctiforme* PCC 73102 and *Anabaena sp.* PCC 7120 (Ishikawa et al. 2023). Analysis of the *Δslr2103* mutant and an *slr2103* overexpression lines in Syn6803 and *Synechococcus sp.* PCC 7942 (Syn7942), indicated that slr2103 is involved in the synthesis of acyl-PQH and acyl-PQC (Ishikawa et al. 2023; Kondo et al. 2023b; Mori-Moriyama et al. 2023).

The slr2103-like sequences are widely distributed among cyanobacteria. *Synechococcus sp.* PCC 7002 (Syn7002) which contains the *slr2103*-related gene *SYNPCC7002_A0918* (abbreviated A0918), accumulates acyl-PQH and acyl-PQC, and the *Δa0918* mutant lacks acyl-PQH and acyl-PQC. Syn7942 which lacks an *slr2103*-related gene, does not contain acyl-PQH and acyl-PQC, but expression of *A0918* conferred the synthesis of acyl-PQH and acyl-PQC in strain Syn7942 (Kondo et al. 2023a). On the other hand, conflicting results were obtained on the presence of TAG and FAPE, because these two lipids were identified in Syn6803 (Aizouq et al. 2020), but not in other studies (Ishikawa et al. 2023; Kondo et al. 2023a, b; Mori-Moriyama et al. 2023). These later studies concluded that *slr2103*-like genes in cyanobacteria are involved in the synthesis of acyl-PQH and acyl-PQC rather than TAG and FAPE.

To study the capacity of nonpolar lipid synthesis in cyanobacteria and to elucidate the involvement of *slr2103*-like genes, we re-evaluated the synthesis of nonpolar lipids in Syn6803 and Syn7002 which contain the acyltransferase genes *slr210*3 and *A0918*, respectively, as well as in the strain Syn7942 which lacks an *slr2103*-related gene. We also included *Gloeobacter violaceus* PCC 7421 (Glo7421) which also lacks an *slr2103*-related gene. Glo7421 is a primitive cyanobacterium, which has no thylakoids and the photosystems are localized to the plasma membrane. We compared the accumulation of TAG, FAPE, acyl-PQH and acyl-PQC in wild-type and mutant cyanobacterial strains, and analyzed acyltransferase activity of slr2103 and A0918 proteins with different substrates after expression in *E. coli* and in the respective mutants. These experiments revealed that *slr2103*-related genes encode multifunctional acyltransferases (MFAT) which are capable of synthesizing a wide array of nonpolar lipids including TAG, FAPE, acyl-PQH and acyl-PQC in different cyanobacteria.

## Materials and methods

### Cells and growth conditions

Cells of *Synechocystis sp.* PCC 6803 (Syn6803), *Synechococcus sp.* PCC 7002 (Syn7002), *Synechococcus sp.* PCC 7942 (Syn7942), and *Gloeobacter violaceus* PCC 7421 (Glo7421) were obtained from the Pasteur Culture Collection of cyanobacteria (Paris, France). Cells were grown on BG-11 medium in flasks with low agitation (100 rpm) at 25 ℃ and incessant light of 25 µmol m^−2^ s^−1^. Cells were grown at low light intensity because they suffered from bleaching at light intensities > 50 µmol m^−2^ s^−1^. The pH was around 7.3 at the time of inoculation. The medium was in exchange with CO_2_ of the ambient air. For Glo7421, the medium was supplemented with 10 mM sodium bicarbonate. For Syn7002, Turk Island Salts (10%, v/v; Medium 1550 in Pasteur Culture Collection), 10 mM glycerol and vitamin B12 (4 μg/l) were added to the medium (Lambert and Stevens 1986).

### Construction of the *Δslr2103* mutant of *Synechocystis sp. *PCC 6803 and the *Δa0918* mutant of *Synechococcus sp.* PCC 7002

The *Δslr2103* mutant of Syn6803 was described previously (Aizouq et al. 2020). The Syn7002 gene *A0918* (locus SYNPCC7002_A0918*,* Genbank entries WP_012306546, ACA98922) was previously annotated as'PlsC'in Genbank. A0918 is closely related to slr2103 from Syn6803 (Kondo et al. 2023a), but not to sll1752 and sll1848 which harbor LPAAT activity analogous to PlsC from *E. coli* (Weier et al. 2005; Okazaki et al. 2006). The *Δa0918* mutant of Syn7002 was produced by homologous recombination. The 5’ flanking region of A0918 was PCR amplified from genomic DNA with the oligonucleotides Bn4917 and Bn4918 (with *Bsa*I sites), and the 3’ flanking region was amplified with Bn4919 and Bn4920 (with *Bbs*I sites) (Table S1). The kanamycin resistance cassette was amplified with the oligonucleotides Bn3421 and Bn3660 (with *Bsa*I sites). The amplicons were digested with *Bsa*I, *Bbs*I and ligated in a single step into the vector pJ-GG-lacZ (digested with *Bsa*I) to yield the knockout plasmid pJ-Δa0918-Kan. The plasmid was linearized with *Esp*3I and used for natural transformation of Syn7002 (Stevens and Porter 1980; Ruffing 2014). After transformation, cells were selected on medium with increasing kanamycin content (final concentration 50 µg ml^−1^). The integration of the kanamycin resistance cassette into the *A0918* locus by double cross-over was confirmed by PCR of genomic DNA with the oligonucleotides Bn5335/Bn5336 and Bn5337/Bn5338 (Fig. S1).

### Expression of *slr2103* from *Synechocystis sp. *PCC 6803 and *A0918* from *Synechococcus sp. *PCC 7002 in *Escherichia coli*

The cloning of the gene *slr2103* into pQE-80L (Qiagen) and expression in *E. coli* was described previously (Aizouq et al. 2020). The gene *A0918* was amplified from genomic DNA of Syn7002 by PCR using the oligonucleotides Bn4756, Bn4954 (Table S1) and ligated into pJET1.2 (Thermo Fisher Scientific). The *A0918* gene was excised with *Bsa*I and *Pst*I and cloned into pQE-80L (Qiagen). *E. coli* cells (ElectroSHOX, Bioline) were grown in LB medium at 37 ℃ to an optical density OD_600_ of 0.5–0.6. Protein expression was induced with 0.5 mM isopropyl-β-D-thiogalactopyranoside (IPTG) and the cells incubated at 16 ℃ overnight. Protein expression was confirmed by SDS polyacrylamide gel electrophoresis with Coomassie Brilliant Blue staining (Fig. S1).

### Determination of acyltransferase activity of slr2103 and A0918 after expression in *E. coli*

*E. coli* cells were grown at 37 ℃ to an OD_600_ of 0.5–0.6 before induction with 0.5 mM IPTG (Aizouq et al. 2020). The cells from a 50 ml culture were harvested, suspended in 5 ml of medium and supplemented with 1500 nmol of one of the substrates, dioctanoin (di8:0 diacylglycerol, dioctanoylglycerol, SigmaAldrich/Merck), phytol (Chemimpex), or decylplastoquinone (DPQ; Santa Cruz Biotechnology) (all substrates dissolved in a minimal volume of toluene). The cultures were incubated at 30 ℃ with agitation at 220 rpm for 3 h. Cells were harvested, washed twice with water and the OD_600_ was determined, prior to lipid extraction.

For enzyme assays, protein expression was induced with 0.5 mM IPTG during growth at 16 ℃ overnight. The cells were harvested and suspended in homogenization buffer (1 mM EDTA, 200 mM sucrose, 100 mM Tris–HCl, pH 7.4) and homogenized with a sonicator. The cell extract was centrifuged at 10,000×*g* for 2 min, and the supernatant was centrifuged at 28,000×*g* for 1 h. The pellet (microsomal membranes) was resuspended in homogenization buffer, and the protein concentration was measured with bicinchoninic acid. The acyltransferase assay contained 50 μM palmitoyl-CoA (16:0-CoA), 200 μM substrate (phytol, dioctanoin, or DPQ; dissolved in ethanol) and 400 μg of the microsomal protein fraction in the assay buffer (20 mM MgCl_2_, 0.1% CHAPS, 100 mM Tris–HCl, pH 7.4, 1.25 mg ml^−1^ BSA, 10 mM Na orthovanadate). The final reaction volume was 200 μl. The reaction was mixed and incubated for 20 min at 37 ℃. The reaction was terminated and lipids extracted by adding 1 ml of methyl tert-butyl ether and 300 µl methanol.

### Supplementation of decylplastoquinone to *Synechococcus* sp. PCC 7002 WT and *Δa0918* mutant

Syn7002 WT and *Δa0918* cultures (100 ml) grown under standard conditions to an OD_730_ of 0.7 were harvested, suspended in 1 ml of fresh medium (containing kanamycin for the mutant) and supplemented with 300 nmol of DPQ (in toluene). The cultures were incubated at 25 ℃ with agitation (110 rpm) under standard light conditions for 4 days. Cells were harvested, washed twice with water, and the OD_730_ was determined followed by lipid extraction.

### Lipid isolation and separation

Lipids were extracted from cyanobacterial or *E. coli* cells, or from in vitro enzyme assays as described (Matyash et al. 2008). Briefly, the cells were incubated in 200 µl of water at 100 ℃ for 15 min to inactivate enzyme activities. Then 0.3 ml of methanol, 1 ml of methyl tert-butyl ether and internal standards were added. The mixture was placed on a shaker for 1 h. Water (0.25 ml) was added, and the mixture was centrifuged to obtain phase separation. The upper phase was collected, dried with nitrogen gas and lipids dissolved in hexane.

Nonpolar lipids were purified by solid phase extraction (SPE) on silica columns (Chromabond, 500 mg silica; Macherey & Nagel) equilibrated with hexane. The lipid extract dissolved in hexane was loaded on the column. FAPEs were eluted with hexane/diethyl ether (99:1, v/v). TAGs and the acylated plastoquinol lipids (acyl-PQH, acyl-PQC, acyl-DPQ) were eluted with hexane/diethyl ether (95:5, v/v).

Lipids were separated by thin layer chromatography (TLC) on silica plates (Silica 60 Durasil, Macherey & Nagel) with hexane, diethyl ether, acetic acid (70:30:1, by vol.). Lipids co-migrating on the TLC plates were stained with primuline and visualized under UV light (White et al. 1998). The lipids were isolated from the silica material with methyl tert-butyl ether/methanol (1:0.3, v/v) for further analysis.

### Lipid measurement by mass spectrometry

The eluted lipids were dried under a nitrogen gas stream. TAG and FAPE were dissolved in chloroform/methanol/300 mM ammonium acetate (300:665:35, by vol.) and measured by direct infusion nanospray MS/MS on an Agilent 6530 Q-TOF instrument (Aizouq et al. 2020). The internal standards for TAG and FAPE were tri17:0-TAG/tri17:1-TAG (Larodan) and 17:0-phytol, respectively.

Acylated plastoquinols (acyl-PQH, acyl-PQC, acyl-DPQ) were dissolved in tetrahydrofuran/methanol/5 mM ammonium formate (7:2:1, by vol., 0.1% formic acid), and quantified by LC–MS/MS. Acyl-PQH and acyl-PQC were measured as relative peak areas, and acyl-DPQ was measured relative to 17:0-DPQ. The acylated plastoquinols were separated by LC–MS/MS on a Supelcosil ABZ plus column (3 μm, 10 cm × 2.1 mm i.d., SigmaAldrich/Merck) using a gradient elution of solvent A (tetrahydrofuran/methanol/5 mM ammonium formate, 3:2:5, by vol., 0.1% formic acid) and solvent B (tetrahydrofuran/5 mM ammonium formate, 9:1, v/v, 0.1% formic acid). The flow rate was 0.3 ml min^−1^ and the gradient was set as: 0 min, 60% B; 8 min, 100% B; 15 min, 100% B; 15.1 min, 60% B and 20 min, 60% B. The ion source was operated in the positive mode.

Lipid standards (17:0-phytol, 17:0-DPQ) were synthesized as described (Gellerman et al. 1975). Briefly, the fatty acid was converted into the acyl chloride with oxalyl chloride. The esterification reaction was carried out with the acyl chloride and phytol or DPQ (which had been reduced with NaBH_4_). The internal standards were purified by SPE on silica columns and quantified by gas chromatography of fatty acid methyl esters (Browse et al. 1986).

### Transmission electron microscopy

Transmission electron microscopy was done at the Microscopy Core Facility of University of Bonn as described (Aizouq et al. 2020). The cells were fixed in fixation buffer containing 3% NaCl, 4% paraformaldehyde, 2.5% glutaraldehyde in 0.1 M cacodylate buffer. After washing with 0.1 M cacodylate buffer, cells were treated in 1% OsO_4_ and 0.8% ferricyanate in cacodylate buffer for 2 h. Cells were embedded in 1% melted agarose, solidified on ice, the agarose blocks trimmed and dehydrated with an ethanol series (30%, 50%, 70%). The blocks were incubated in 0.5% uranyl acetate in 70% ethanol for 1 h. Finally, the blocks were further dehydrated with ethanol (90%, 95%, 100%) and propylene oxide, and the samples were infiltrated with EPON epoxy resin (Merck/Sigma-Aldrich). Resin-embedded blocks were cured at 60 °C for 48 h. Ultrathin sections (~ 50 nm) were cut using an ultramicrotome and collected on Formvar/carbon-coated TEM Copper Slot Grids (Science Services, Munich). Finally, transmission micrographs were observed using a Crossbeam 550 electron microscope (Zeiss, Oberkochen) at 30 kV acceleration voltage and 150 pA beam current with a STEM (scanning transmission electron microscopy) detection high resolution mode.

## Results

### Isolation of TAG and FAPE from cyanobacteria

We previously showed that Syn6803 accumulates TAG and FAPE, while the *Δslr2103* mutant of Syn6803 carrying a mutation in the acyltransferase gene *slr2103* was deficient in TAG and FAPE (Aizouq et al. 2020). The two lipids were detected in Syn6803 cells by mass spectrometry after purification via SPE on silica columns. Recently, two acylated forms of plastoquinol (acyl-PQH, acyl-PQC) were detected in Syn6803 which were absent from the *Δslr2103* mutant, indicating that slr2103 is also involved in the synthesis of acyl-PQH and acyl-PQC (Ishikawa et al. 2023; Kondo et al. 2023a, b; Mori-Moriyama et al. 2023). In these studies, the nonpolar lipids were separated by TLC, and lipids co-migrating with a TAG standard were isolated. Only acyl-PQH and acyl-PQC, but not TAG and FAPE, were detected after analysis by mass spectrometry.

**Fig. 1:**
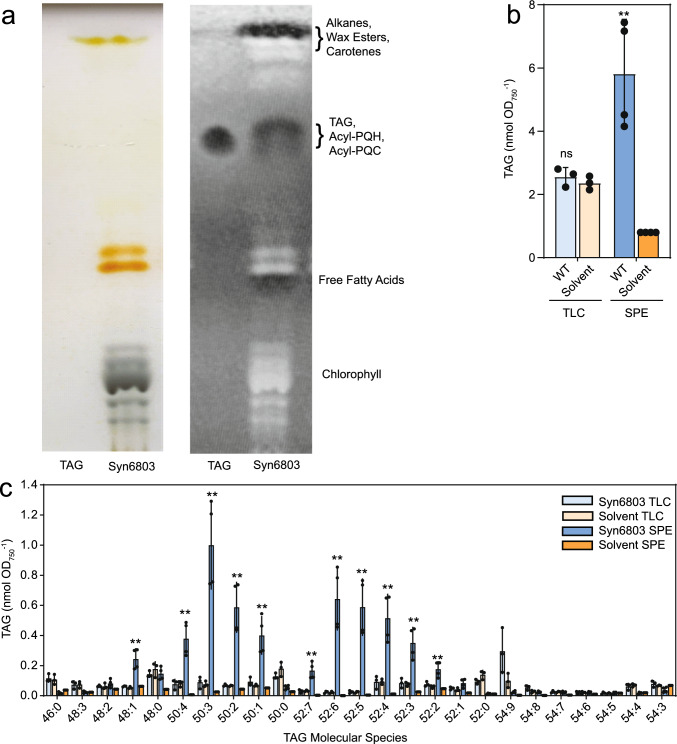
Isolation of nonpolar lipids from *Synechocystis sp.* PCC 6803. TAG was isolated from Syn6803 cells by thin-layer chromatography (TLC) or solid phase extraction (SPE) prior to measurements by mass spectrometry. **a** TLC plate showing the migration of TAG. The plate was photographed (left) or observed under UV light (right) for lipids stained with primuline. **b** Total TAG content in lipid extracts from Syn6803 cells and in a solvent control sample, after isolation by TLC or SPE. **c** Molecular species composition of TAG. Mean values ± SD; *n* = 3; Student T test; significant differences to the respective solvent control; *P < 0.05; **P < 0.01; *ns* not significant

When we employed the TLC protocol for the isolation of TAG from Syn6803, only a low amount of TAG (~ 2.5 nmol OD_750_^−1^) was found which was similar to the amount in the solvent control (~ 2.4 nmol OD_750_^−1^) (Fig. 1). This result indicates that the solvent control contains TAG derived from contamination in the TLC plate or solvents used for chromatography, and that the TAG from Syn6803 cells was lost during TLC purification (Fig. 1). The TAG contaminants in the Syn6803 sample and the solvent control after TLC purification were mostly saturated (48:0, 50:0, 52:0, 54:0) except for 54:9. However, when lipids were isolated by SPE, the amount of TAG in the solvent control was lower (~ 0.8 OD_750_^−1^), and the amount of TAG from the Syn6803 cells was ~ 8 times higher (~ 6 nmol OD_750_^−1^) (Fig. 1). SPE-purified TAG from Syn6803 cells contained mostly unsaturated molecular species (50:4, 50:3, 50:2, 50:1, 52:6, 52:5, 52:4, 52:3), which were very low in the solvent control. These results indicate that the silica columns and solvents used for SPE are largely free of TAG contamination, and that the losses of TAG from Syn6803 cells during SPE chromatography are minimal.

### Accumulation of nonpolar lipids in *Synechocystis sp. *PCC 6803, *Synechococcus sp. *PCC 7002, *Synechococcus sp. *PCC 7942 and *Gloeobacter violaceus* PCC 7421

**Fig. 2:**
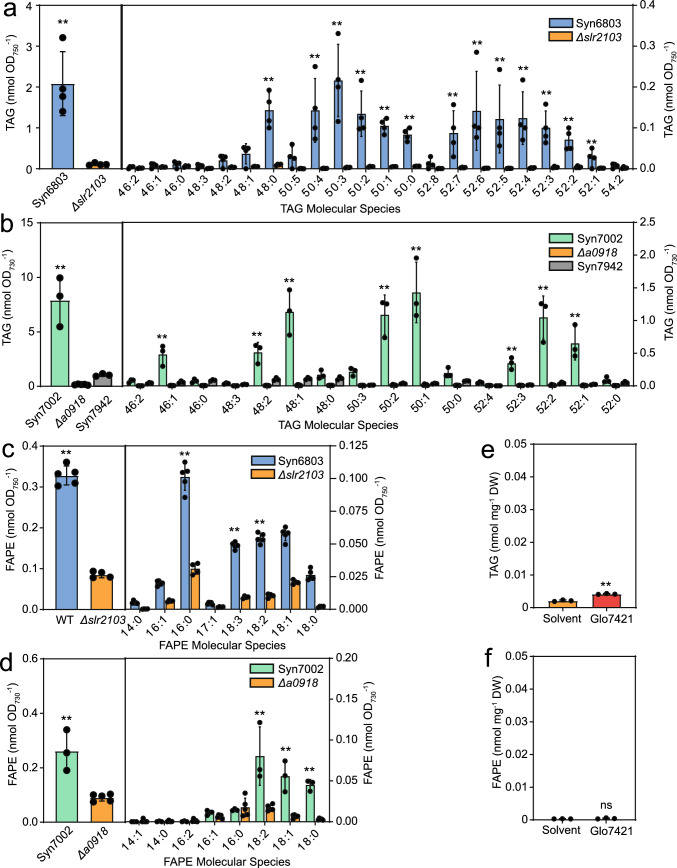
Accumulation of triacylglycerol and fatty acid phytyl esters in *Synechocystis sp.* PCC 6803, *Synechococcus sp.* PCC 7002, *Synechococcus sp.* PCC 7942 and *Gloeobacter violaceus* PCC 7421. Nonpolar lipids were purified by SPE and measured by direct infusion MS/MS (TAG, FAPE) and LC–MS/MS (acyl-PQH, acyl-PQC). **a** TAG content and composition in Syn6803 and in the *Δslr2103* mutant. **b** TAG content and composition in Syn7002, the *Δa0918* mutant, and Syn7942. **c** FAPE content and composition in Syn6803 and in the *Δslr2103* mutant. **d** FAPE content and composition in Syn7002 and the *Δa0918* mutant. **e** TAG and **f** FAPE contents in Glo7421. The molecular species of TAG and of FAPE in Glo7421 were at the detection limit. Mean values ± SD; *n* = 3–5; Student T test; significant differences between WT and mutant or solvent control; *P < 0.05; **P < 0.01; *ns* not significant

We employed the SPE protocol to purify nonpolar lipids from Syn6803 and the *Δslr2103* mutant. The total amount of TAG was ~ 20-fold higher in Syn6803 compared with the *Δslr2103* mutant, and the Syn6803 TAG contained mostly unsaturated molecular species (Fig. 2a) in agreement with previous results (Aizouq et al. 2020).

We next measured TAG after SPE purification in Syn7002 cells, which contain the *slr2103*-like gene *A0918*, and in the corresponding *Δa0918* mutant. Furthermore, Syn7942 was included which lacks an *slr2103*-like gene. Considerable amounts of TAG were detected in Syn7002, while it was very low in the *Δa0918* mutant and in Syn7942 (Fig. 2b). TAG in Syn7002 contained mostly unsaturated molecular species with 1 to 3 double bonds. The TAG molecular species of Syn6803 were much more unsaturated compared with Syn7002, in line with the finding that Syn6803 contains higher amounts of polyunsaturated fatty acids (6% 18:2; 29% 18:3; 8% 18:4; at 22 ℃) compared with Syn7002 (25% 18:2; 10% 18:3; at 22 ℃)(Wada and Murata 1990; Sakamoto et al. 1997). Presumably, the acyl composition of the total membrane lipids is reflected in the TAG molecular species composition.

The amount of FAPEs measured by mass spectrometry after SPE purification was ~ fourfold higher in Syn6803 compared with the *Δslr2103* mutant (Fig. 2c), in accordance with previous measurements (Aizouq et al. 2020). FAPEs in Syn6803 mostly contained 16:0 and unsaturated C18 fatty acids. Similarly, the FAPE content in Syn7002 was ~ 2.5 fold higher compared with the *Δa0918* mutant (Fig. 2d). In Syn7002, the FAPEs contained high amounts of 18:0, 18:1 and 18:2 acyl groups. *Gloeobacter violaceus* PCC 7421 (Glo7421) is a primitive cyanobacterium that is devoid of *slr2103*-like genes, similar to Syn7942. The amounts of TAG and FAPE in Glo7421 were extremely low, and they were slightly above, or similar to the solvent control level, respectively (Fig. 2e, g). These results indicate that slr2103 and A0918 are involved in the synthesis of TAG and FAPE with 16:0, 18:0 and unsaturated C18 fatty acids in Syn6803 and Syn7002, respectively. Syn7942 and Glo7421 which are devoid of an slr2103-like gene contain only low amounts of TAG or FAPE.

### Synthesis of acyl-PQH and acyl-PQC in *Synechocystis sp. *PCC 6803, *Synechococcus sp. *PCC 7002, *Synechococcus sp. *PCC 7942 and *Gloeobacter violaceus* PCC 7421

**Fig. 3:**
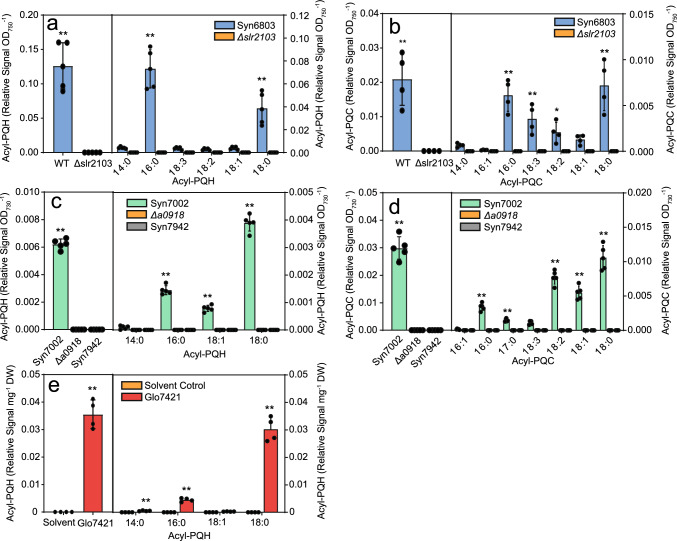
Contents and composition of acyl-PQH and acyl-PQC in *Synechocystis sp*. PCC 6803, *Synechococcus sp.* PCC 7002, *Synechococcus sp.* PCC 7942 and *Gloeobacter violaceus* PCC 7421. Acylated plastoquinols were isolated by SPE and the peak areas were measured by LC–MS/MS as relative signals. **a** Acyl-PQH and **b** acyl-PQC contents and composition in the Syn6803 and the *Δslr2103* mutant. **c** Acyl-PQH and **d** acyl-PQC contents and composition in Syn7002, the *Δa0918* mutant and Syn7942. **e** Acyl-PQH content and composition in Glo7421. Note that no acyl-PQC esters were detected in Glo7421. Mean values ± SD; *n* = 4–5; Student T test; significant differences between WT and mutant or solvent control; *P < 0.05; **P < 0.01; *ns* not significant

We measured acyl-PQH and acyl-PQC in the cyanobacteria after purification by SPE. The signals of total acyl-PQH in Syn6803 were higher compared with acyl-PQC, and the two forms of acylated plastoquinols were absent from the *Δslr2103* mutant (Fig. 3a, b), in line with previous results (Ishikawa et al. 2023; Kondo et al. 2023a, b; Mori-Moriyama et al. 2023). Saturated acyl groups (16:0, 18:0) were most abundant in acyl-PQH and acyl-PQC, but acyl-PQC also contained unsaturated fatty acids (18:1, 18:2, 18:3). Unsaturated acyl-PQC esters were previously not detected in Syn6803 (Ishikawa et al. 2023; Kondo et al. 2023a, b; Mori-Moriyama et al. 2023).

Syn7002 was previously shown to produce acyl-PQH and acyl-PQC, while these two acylated plastoquinols were absent from the *Δa0918* mutant and from Syn7942 (Kondo et al. 2023a). Measurements by LC–MS/MS of lipids isolated by SPE confirmed these results (Fig. 3c, d). The acylated plastoquinols contained mostly 18:0, and lower amounts of 16:0 and unsaturated fatty acids, and the acyl groups of acyl-PQC were more unsaturated compared with acyl-PQH (Fig. 3c, d).

Acyl-PQH was also detected in Glo7421 in higher amounts compared with the solvent control (Fig. 3e). Only saturated acyl groups (16:0, 18:0) were detected in acyl-PQH of Glo7421, but no acyl-PQC ws found. Therefore, acyl-PQH esters can also occur in cyanobacteria like Glo7421 which are devoid of an *slr2103*-like gene.

### TAG, FAPE and acyl-DPQ synthesis activities of slr2103 and A0918

**Fig. 4:**
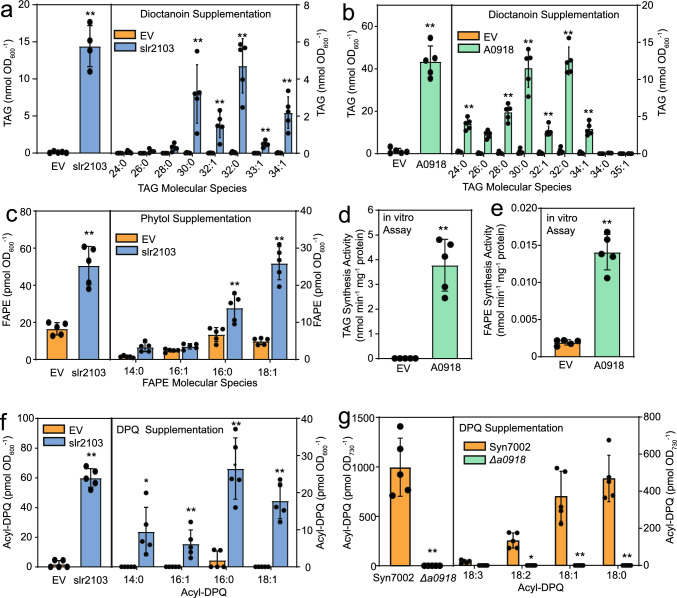
Acyltransferase activity of slr2103 and A0918 proteins. Supplementation of *E. coli* cells expressing slr2103 from Syn6803 or A0918 from Syn7002, or of Syn7002 WT and *Δa0918* cells with dioctanoin, phytol or decylplastoquinone (DPQ) results in the accumulation of TAG, FAPE, or acyl-DPQ, respectively. **a** Accumulation of TAG after supplementation of *E. coli* cells expressing slr2103 with dioctanoin. **b** Accumulation of TAG after supplementation of *E. coli* cells expressing A0918 with dioctanoin. **c** Accumulation of FAPE after supplementation of *E. coli* cells expressing slr2103 with phytol. **d** In vitro assay of A0918 expressed in *E. coli* with dioctanoin. **e** In vitro assay of A0918 expressed in *E. coli* with phytol. **f** Accumulation of acyl-DPQ after supplementation of *E. coli* cells expressing slr2103 with DPQ. **g** Accumulation of acyl-DPQ after supplementation of Syn7002 WT and *Δa0918* cells with DPQ. Mean values ± SD; *n* = 4–5; Student T test; significant differences between EV (empty vector control) and *slr2103* or *A0918* expressing cells, or Syn7002 WT and *Δa0918*; *P < 0.05; **P < 0.01

We tested whether *E. coli* cells expressing slr2103 or A0918 have the capacity to acylate nonpolar lipid substrates. The acylated products were measured by mass spectrometry after SPE purification. As previously shown, TAG and FAPE were synthesized after supplementation of *E. coli* cells expressing *slr2103* with dioctanoin or phytol, respectively (Fig. 4a, c) (Aizouq et al. 2020). Supplementation of the *E. coli* cells expressing *A0918* with dioctanoin also resulted in TAG accumulation (Fig. 4b). The molecular species of TAG and FAPE produced in *E. coli* expressing *slr2103* and of TAG from *A0918* expressing cells, contained acyl groups typically found in *E. coli*, including 14:0, 16:0, 16:1Δ9 and 18:1Δ11 (Fig. 4a, b, c). We next performed an in vitro assay with A0918 protein extracted from recombinant *E. coli* cells using dioctanoin or phytol in combination with palmitoyl-CoA as substrates. TAG and FAPE were produced in these in vitro assays, confirming that A0918 harbors acyltransferase activity for the synthesis of TAG and FAPE (Fig. 4d, e).

Because authentic plastoquinol-9 or plastoquinol-9 carrying a hydroxyl group on the isoprenoid chain are not commercially available, we employed decylplastoquinol (DPQ), an analog of plastoquinol-9, for acyltransferase assays. *E. coli* cells expressing slr2103 were supplemented with DPQ, lipids purified by SPE and measured by LC–MS/MS. Figure 4f shows that slr2103 produced acyl-DPQ with fatty acids derived from *E. coli*. These results confirm that slr2103 is capable of acylating one of the quinol hydroxyl groups of DPQ. Attempts to measure acylation of DPQ with the A0918 protein expressed in *E. coli* were not successful. Therefore, Syn7002 WT and *Δal0918* cells were supplemented with DPQ. After four days, lipids were extracted, and acyl-DPQ was measured by LC–MS/MS. The Syn7002 WT produced acyl-DPQ, mostly containing 18:2, 18:1 and 18:0, in contrast to the *Δa0918* mutant, confirming that A0918 is capable of acyl-DPQ synthesis (Fig. 4f).

## Discussion

Under adverse physiological conditions, cyanobacteria change their metabolism and store excess carbon in the form of glycogen and polyhydroxybutyrate. On the other hand, plants and eukaryotic algae store excess carbon mostly in the form of starch, a glycogen-like glucose polymer, and TAG. The presence of TAG in cyanobacteria was controversial, but it was confirmed in Syn6803 cells (Figs. 1, 2a), and the gene *slr2103* was found to be required for TAG synthesis (Aizouq et al. 2020). Four classes of nonpolar lipids, including TAG, FAPE, acyl-PQH and acyl-PQC (PQB) were found in Syn6803 and other cyanobacteria. Recently, a plastoquinol C form carrying two acyl groups at the quinol hydroxyl and isoprenoid hydroxyl groups (diacyl-PQC; acyl-PQB) was identified in Syn6803 and other cyanobacteria, but it was absent from the *Δslr2103* mutant (Tanikawa et al. 2025). We calculated the total amounts of membrane lipids (29.1 µmol OD_750_^−1^), TAG (2.19 nmol OD_750_^−1^) and FAPE (0.32 nmol OD_750_^−1^) in Syn6803 cells. Syn7002 cells contain 14.2 µmol OD_750_^−1^ of total membrane lipids, 7.9 nmol OD_750_^−1^ of TAG and 0.25 nmol OD_750_^−1^ of FAPE. Therefore, the TAG and FAPE contents relative to total membrane lipids in these cyanobacteria are very low. The amounts of the acylated plastoquinol lipids are presumably also low, but their absolute quantification was not possible due to the lack of authentic standards. The acyl groups and phytol for TAG and FAPE synthesis are most likely derived from the degradation of membrane lipids and chlorophyll, respectively, while plastoquinol originates from the photosynthetic membranes. The function of the nonpolar lipids remains enigmatic. The growth of *Δslr2103* under optimal conditions was very similar in comparison with WT (Aizouq et al. 2020). Previously, the *Δa0918* mutant cells were found to display a growth rate similar to WT in a bubble culture, but they grew slightly slower in a static culture (Kondo et al. 2023a). Therefore, the *MFAT* genes are not essential during normal growth, but might be important during stress conditions, as they might be involved in the sequestration of acyl groups and phytol from membrane lipids and chlorophyll, respectively, and in the homeostasis of the photosynthetically active plastoquinol pool under stress. It is difficult to study the function of the individual nonpolar lipids by analyzing the *Δslr2103* or *Δa0918* mutants, because the mutants are simultaneously deficient in all of the nonpolar lipids.

Divergent results were obtained regarding the occurrence of TAG and FAPE in Syn6803 and other cyanobacteria (Ishikawa et al. 2023; Kondo et al. 2023b; Mori-Moriyama et al. 2023). When nonpolar lipids were isolated by TLC prior to LC–MS analysis, only acyl-PQH and acyl-PQC were identified, but not TAG and FAPE. In particular, the unsaturated molecular species of TAG (and of acyl-PQH and acyl-PQC) were lost, such that only saturated TAG species were detected in amounts similar to background (Ishikawa et al. 2023; Kondo et al. 2023a, b; Mori-Moriyama et al. 2023). During TLC separation, the lipids are exposed to oxygen in the air when the plates are dried after chromatography. Unsaturated acyl groups are oxidized when exposed to air after drying the plate, and this oxidation is even more pronounced when minute amounts of lipids are separated (Fuchs et al. 2009; Zhou et al. 2014). Furthermore, TAG can be present as a contaminant in solvents or the TLC plates, giving rise to background signals during mass spectrometry. On the other hand, direct analysis of TAG extracted from cyanobacteria by LC–MS/MS without prior enrichment circumvents the losses during TLC separation, but can result in ion suppression due to the very low content of TAG compared with membrane lipids. Indeed, TAG was identified in Syn6803 when using LC–MS analysis without prior TLC separation (Ishikawa et al. 2023; Tanikawa et al. 2025). TAG was also detected in Syn7002 (Kondo et al. 2023a). The technical problems of enrichment of nonpolar lipids can be overcome by using SPE purification before analysis by mass spectrometry (Fig. 1) (Aizouq et al. 2020). During SPE the lipids always remain covered with solvent such that they are not exposed to the air.

FAPE was not detected in the lipid spot co-migrating with TAG during TLC separation in the previous studies (Ishikawa et al. 2023; Mori-Moriyama et al. 2023). This can be explained because FAPEs do not co-migrate with TAG as they are more nonpolar, instead they co-migrate with wax esters close to the solvent front during TLC separation (Fig. 1) (Ischebeck et al. 2006). Using SPE for lipid purification, we found considerable amounts of TAG and FAPE in Syn6803 and Syn7002 cells, but not in the *Δslr2103* and *Δa0918* mutants, confirming that the genes *slr2103* and *A0918* are responsible for producing TAG and FAPE.

We confirmed that Syn6803 and Syn7002 contain acyl-PQH and acyl-PQC, while the *Δslr2103* and *Δa0918* mutants are deficient in these acylated plastoquinols. In contrast to the previous studies, we detected unsaturated acyl groups in the acylated plastoquinols (Fig. 3a–d). This difference can be explained by the use of SPE for lipid purification prior to LC–MS/MS, because unsaturated molecular species are preferably lost during TLC purification (Ishikawa et al. 2023; Kondo et al. 2023a, b; Mori-Moriyama et al. 2023). The proportion of unsaturated acyl groups was higher in acyl-PQC compared with acyl-PQH. Therefore, a higher proportion of acyl groups in acyl-PQC is presumably derived from membrane lipids which in general are more highly unsaturated than de novo synthesized fatty acids.

We previously observed differences in the number of electron dense structures tentatively identified as lipid droplets between the WT and *Δslr2103* mutant of Syn6803 (Aizouq et al. 2020). This could be explained by the lack of the nonpolar lipids TAG, FAPE and acylated forms of plastoquinol in the *Δslr2103* mutant. Analysis by transmission electron microscopy of *Syn7002* WT and *Δa0918* mutant cells demonstrated that the cells were similar, and we could not find lipid droplets (Fig. S2) (Gonzalez-Esquer et al. 2016). Therefore, the occurrence of lipid droplets might depend on the species and growth conditions. The accumulation of TAG and FAPE in Syn6803 and Syn7002 cells depends on the acyltransferase genes *slr2103* and *A0918*, respectively, because the corresponding mutants are devoid of TAG and FAPE (Fig. 2a–d) (Aizouq et al. 2020). These results were confirmed by expressing *slr2103* and *A0918* in *E. coli*, because the two acyltransferases produced TAG from dioctanoin and FAPE from phytol (Fig. 4a–e) (Aizouq et al. 2020).

The acyltransferases slr2103 from Syn6803 and A0918 from Syn7002 are also involved in the synthesis of acyl-PQH and acyl-PQC as revealed by the presence of acylated plastoquinol lipids in Syn6803 and Syn7002 WT, and the absence in *Δslr2103* and *Δa0918* mutant cells as well as in Syn7942 which lacks an *slr2103*-like gene (Fig. 3a–d) (Ishikawa et al. 2023; Kondo et al. 2023b; Mori-Moriyama et al. 2023). We used the plastoquinol-9 analog DPQ which carries the same quinol head group but has a shorter side chain, for supplementation experiments, and identified acyl-DPQ esters in the *E. coli* cells expressing *slr2103* (Fig. 4f). As heterologously expressed A0918 protein was not able to acylate DPQ, we confirmed its acylation activity by supplementing DPQ to Syn7002 WT and *Δa0918* mutant cells. These results demonstrate that DPQ is taken up by the *E. coli* and Syn7002 cells and reduced to decylplastoquinol, presumably by enzymes of the menaquinone and ubiquinone metabolism, prior to acylation by slr2103 or A0918. The accumulation of acyl-DPQ in supplementation experiments of *E. coli* cells expressing *slr2103* with DPQ was ~ 60 pmol OD_600_^−1^, which was in the range of FAPE (~ 50 pmol OD_600_^−1^) accumulation after feeding phytol, but much lower compared with the amount of TAG (~ 15 nmol OD_600_^−1^) after dioctanoin supplementation. These results might suggest that the activity of slr2103 is highest for TAG synthesis, because the acyltransferase capacity with the different substrates was in the order dioctanoin > > DPQ ≈ phytol, although it remains unclear whether the uptake and the metabolism by enzymes in *E. coli* might differ among these substrates.

**Fig. 5:**
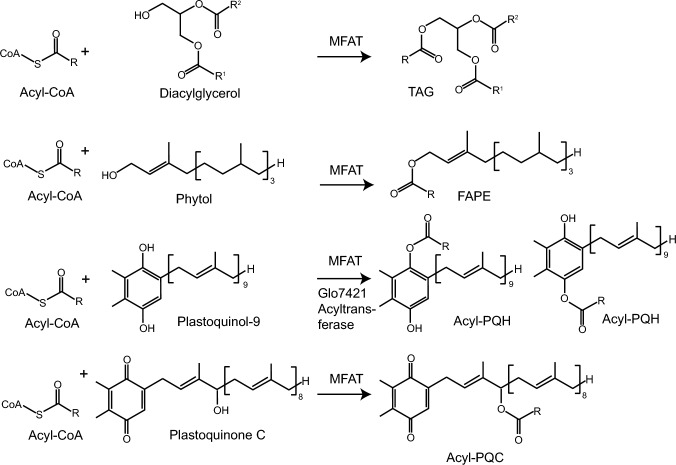
Substrate specificity of multifunctional acyltransferases (MFAT) from cyanobacteria. MFAT acyltransferases accept diacylglycerol, phytol, plastoquinol-9 and plastoquinone C (PQC) as substrates producing triacylglycerol (TAG), fatty acid phytyl esters (FAPE), acyl-plastoquinol-9 (acyl-PQH, acylation of either quinol hydroxy group) and acyl-plastoquinone C (acyl-PQC)

Taken together, slr2103 and A0918 are active with a broad range of hydroxyl-group containing substrates, because these enzymes can acylate diacylglycerol, phytol, the quinol groups of plastoquinol and the hydroxyl group on the isoprenoid chain of hydroxy-plastoquinol (PQC), indicating that they represent multifunctional acyltransferases (MFAT) (Fig. 5). The acyl donor in the in vitro enzyme assays was palmitoyl-CoA. It is possible, that in vivo, acyl-CoA or acyl-(acyl carrier protein) (acyl-ACP) thioesters are the substrates for the acylation reactions.

The two cyanobacterial strains, Syn7942 and Glo7421, are devoid of *MFAT*-like genes. While the amounts of TAG and FAPE were very low in Syn7942 and Glo7421, cells of Glo7421 but not Syn7942 accumulated acyl-PQH esters containing mostly 16:0 and 18:0. A recent manuscript (Tanikawa et al. 2025) also described that Syn7942 was very low in TAG, acyl-PQH and acyl-PQC, and that acyl-PQH, but not acyl-PQC, were detected in Glo7421. Therefore, Glo7421 can produce acyl-PQH but mostly lacks TAG, FAPE or acyl-PQC, and it employs an acyltransferase which in contrast to the MFAT enzymes displays a very narrow substrate specificity for PQH (Fig. 5). The isolation of the specific acyltransferase from Glo7421 is the subject of current studies. These results emphasize that cyanobacteria produce a multitude of acylated nonpolar lipids using MFAT-related or MFAT-independent pathways.

**Fig. 6:**
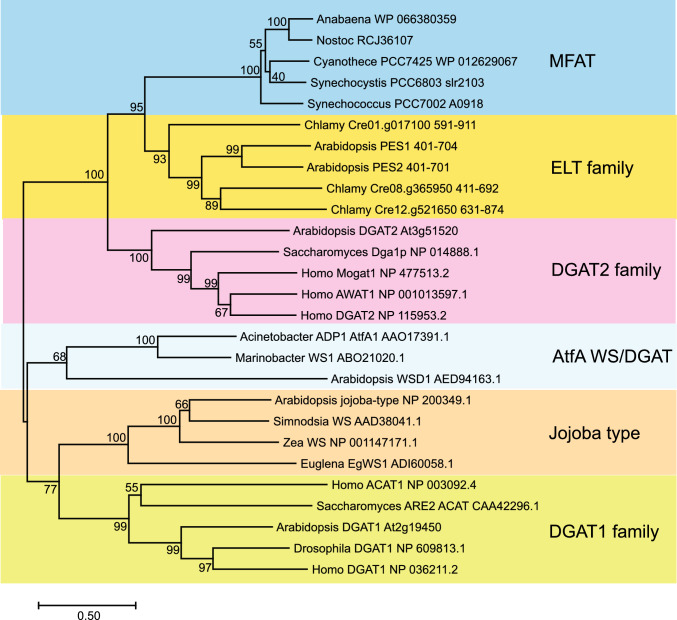
Phylogenetic relationship of acyltransferases involved in nonpolar lipid synthesis. Sequences were aligned with ClustalW, and a phylogenetic tree was built with the Neighbor joining method (bootstrap numbers, 1000) using Mega 11. For the acyltransferases of the ELT family, only the C-terminal amino acids as indicated were used for the alignment

The sequence of slr2103 reveals close similarity with the acyltransferase domain of the ELT (esterase lipase thioesterase) family proteins from plants, with PES1/PES2 (phytyl ester synthases 1/2) from Arabidopsis being functionally characterized (Lippold et al. 2012; Aizouq et al. 2020). Analysis of eukaryotic and prokaryotic acyltransferases confirmed that the MFAT sequences (slr2103 homologs) form a cluster which is a sister group of the PES1- (ELT) like sequences from plants (Mori-Moriyama et al. 2023). On the other hand, the MFAT sequences slr2103 and A0918 were placed into a clade close to eukaryotic DGAT2 sequences (Tanaka et al. 2020; Ishikawa et al. 2023; Kondo et al. 2023b). Figure 6 shows that the cyanobacterial MFAT proteins (including slr2103 and A0918) indeed establish a sister clade with closest sequence similarity to the acyltransferase domain of plant and algal ELT sequences, while they are further apart from eukaryotic (animal, plant, yeast) DGAT2 enzymes and even more distantly related with acyltransferases of the eukaryotic (plant) jojoba type (wax ester synthases), the eukaryotic (animal, plant, yeast) DGAT1 family and the prokaryotic/eukaryotic (bacterial, plant) AtfA, WS/DGAT family. Therefore, the MFAT sequences are presumably the evolutionary precursors for the acyltransferase domain of the plant ELT enzymes and the eukaryotic DGAT2 enzymes. The close similarity of the cyanobacterial MFAT (slr2103, A0918) sequences and the plant ELT (PES1, PES2) sequences is in accordance with the finding that the two acyltransferase families can produce TAG and FAPE, while acyl-PQH and acyl-PQC synthesis has only been confirmed for the MFAT proteins.

## Supplementary Information

Below is the link to the electronic supplementary material.

Supplementary file1 (EPS 13999 KB)

Supplementary file2 (EPS 10994 KB)

Supplementary file3 (DOCX 14 KB)

## Data Availability

All data generated or analyzed during this study are included in this published article and its supplementary information files.

## Abbreviations

DPQ: Decylplastoquinone
FAPE: Fatty acid phytyl ester
Glo7421: *Gloeobacter violaceus sp.* PCC 7421
MFAT: Multifunctional acyltransferase
OD: Optical density
PQC: Plastoquinone C
PQH: Plastoquinol-9
SPE: Solid phase extraction
Syn6803: *Synechocystis sp.* PCC 6803
Syn7002/7942: *Synechochoccus* PCC 7002/7942
TAG: Triacylglycerol
TLC: Thin layer chromatography
WT: Wild type
X, Y: Fatty acids are abbreviated as X:Y, with X indicating the number of carbon atoms, and Y the number of double bonds
