# Supplementary material for: Multifunctional acyltransferases involved in the synthesis of triacylglycerol, fatty acid phytyl esters and plastoquinol esters in cyanobacteria
Source: Planta. 2025 May 2;261(6):123. doi: 10.1007/s00425-025-04700-6 (PMC12048435; doi:10.1007/s00425-025-04700-6)
Supplement: Supplementary file 3 — Supplementary file3 (DOCX 14 KB) [file 425_2025_4700_MOESM3_ESM.docx]

**Multifunctional acyltransferases involved in the synthesis of triacylglycerol, fatty acid phytyl esters and plastoquinol esters in cyanobacteria**

Amita Shajil Das, Arpita Shajil Das, Zishuo Chen, Helga Peisker, Katharina Gutbrod, Georg Hölzl, Peter Dörmann

**Supplementary Information**

**Table S1: Oligonucleotides used in this study**

| Oligo-nucleotide | Sequence (5'-3')  (Restriction sites underlined) | Restric-tion site | Comment |
| --- | --- | --- | --- |
| Bn3421 | GTATATTTGGTCTCtcacgCTGCCGCAAGCACTCAGG | *Bsa*I | Forward primer, amplification of nptII |
| Bn3660 | GTATTTCCGGTCTCTGGGTTCAGAAGAACTCGTCAAGAAG | *Bsa*I | Reverse primer, amplification of nptII |
| Bn4756 | TTTGGTCTCGGATCCATGCCCCTTTTTCC | *Bsa*I | Forward primer, expression of A0918 in *E. coli* |
| Bn4954 | TTTCTGCAGCTAGGGACAACTTTTAGGGCG | *Pst*I | Reverse primer, expression of A0918 in *E. coli* |
| Bn4917 | GTATATTTGGTCTCAACCGCAATGAAAATCAGGCCTAA | *Bsa*I | Forward primer, amplification of 5’ sequence of A0918 |
| Bn4918 | GTATTTATGGTCTCTACCCAGCCACTGGTTTCTACC | *Bsa*I | Reverse primer, amplification of 5’ sequence of A0918 |
| Bn4919 | GTATATTTGAAGACAGCGTGGAAGCTTGTTATCAGCGG | *Bbs*I | Forward primer, amplification of 3’ sequence of A0918 |
| Bn4920 | GTATATTTGAAGACCAGCGTGCTTCTGGCATAACCTGA | *Bbs*I | Reverse primer, amplification of 3’ sequence of A0918 |
| Bn4978 | CAATGTCGCGGAAAATACCCAC | - | Genotyping of Δa0918 |
| Bn4982 | CGAAACCAGTCATACATCATCAT | *-* | Genotyping of Δa0918 |
| Bn4983 | ATGCATCGCAATATTTGGCTCG | *-* | Genotyping of Δa0918 |
| Bn4981 | CTGAGTCAGAACGGATTGTTGC | *-* | Genotyping of Δa0918 |
| Bn5335 | AACTGATCAACCCTAGACAAACC | - | Genotyping of Δa0918 |
| Bn5336 | AACATCGCATCGAGCGAGCAC | - | Genotyping of Δa0918 |
| Bn5337 | ATTGCATCAGCCATGATGGATAC | - | Genotyping of Δa0918 |
| Bn5338 | AATGAACCTCGCTGGCATCGG | - | Genotyping of Δa0918 |
